# Spontaneous cross-species imitation in interactions between chimpanzees and zoo visitors

**DOI:** 10.1007/s10329-017-0624-9

**Published:** 2017-08-16

**Authors:** Tomas Persson, Gabriela-Alina Sauciuc, Elainie Alenkær Madsen

**Affiliations:** Department of Philosophy, Cognitive Science, Lund University, Box 192, 221 00 Lund, Sweden

**Keywords:** Communication, Play, Social cognition, Prosociality

## Abstract

Imitation is a cornerstone of human development, serving both a cognitive function (e.g. in the acquisition and transmission of skills and knowledge) and a social–communicative function, whereby the imitation of familiar actions serves to maintain social interaction and promote prosociality. In nonhuman primates, this latter function is poorly understood, or even claimed to be absent. In this observational study, we documented interactions between chimpanzees and zoo visitors and found that the two species imitated each other at a similar rate, corresponding to almost 10% of all produced actions. Imitation appeared to accomplish a social–communicative function, as cross-species interactions that contained imitative actions lasted significantly longer than interactions without imitation. In both species, physical proximity promoted cross-species imitation. Overall, imitative precision was higher among visitors than among chimpanzees, but this difference vanished in proximity contexts, i.e. in the indoor environment. Four of five chimpanzees produced imitations; three of them exhibited comparable imitation rates, despite large individual differences in level of cross-species interactivity. We also found that chimpanzees evidenced imitation recognition, yet only when visitors imitated their actions (as opposed to postures). Imitation recognition was expressed by returned imitation in 36% of the cases, and all four imitating chimpanzees engaged in so-called imitative games. Previously regarded as unique to early human socialization, such games serve to maintain social engagement. The results presented here indicate that nonhuman apes exhibit spontaneous imitation that can accomplish a communicative function. The study raises a number of novel questions for imitation research and highlights the imitation of familiar behaviours as a relevant—yet thus far understudied—research topic.

**Electronic supplementary material:**

The online version of this article (doi:10.1007/s10329-017-0624-9) contains supplementary material, which is available to authorized users.

## Introduction

By enabling quick and high-fidelity social learning, imitation is regarded as a key mechanism for mediating the cross-generational transfer of knowledge and skills, (e.g. Nielsen 2009). Besides its ‘cognitive’ or ‘learning’ function, imitation accomplishes important social and communicative functions as well, by facilitating social interaction and promoting prosociality (e.g. Užgiris et al. 1989; Eckerman et al. 1989; Eckerman and Stein 1990; Carpenter et al. 2013; Duffy and Chartrand 2015). In the present study we focus on this social–communicative function of imitation, which, unlike imitation learning is less investigated in nonhuman primates^1^. In fact, this has even been claimed to be absent in nonhuman primates based on the observation that, in the context of imitation learning tasks, chimpanzees exhibit lower levels of joint attention, gaze at the experimenter’s face and show ‘over-imitation’ compared to children (Nielsen 2009; Carpenter and Call 2009). The latter captures the fact that children—but not chimpanzees—show a propensity for slavishly copying actions over outcomes, even when the demonstrated actions are irrelevant for achieving a demonstrated outcome. These differences, it has been argued, are to be attributed to the fact that a motivation to be social and promote shared experience lies behind children’s imitative behaviour.

We contend that such claims are grounded on a too narrow definition of the social function of imitation. Evidence from social, developmental and comparative psychology indicates that the latter encompasses both reactive and arguably non-intentional phenomena (such as nonconscious mimicry and imitation-induced prosociality), as well as more proactive and arguably intentional ones (e.g. certain social conformist tendencies or toddlers’ use of imitation for establishing and maintaining social interaction), as detailed below.

Nonconscious mimicry can be defined as the matching of postures, gestures, verbal or facial expressions between interaction partners, without awareness or intent (for a recent review see e.g. Duffy and Chartrand 2015). In nonhuman primates, non-conscious mimicry has been documented in the form of postural congruence (Jazrawi 2000), rapid facial mimicry (as reviewed e.g. by Mancini et al. 2013b), behavioural contagion (e.g. Madsen et al. 2013; Amici et al. 2014 for recent reviews) and interactional synchrony (Yu and Tomonaga 2015). Intriguingly, nonconscious mimicry seems to have a protracted onset in both human and chimpanzee ontogeny, being shown earliest at 3–4 years of age (Carpenter et al. 2013; Madsen et al. 2013; van Schaik and Hunnius 2016).

Nonconscious mimicry, while not being intentionally communicative, still seems to communicate information that leads to prosociality in both human and nonhuman primates. When mimicked, human adults are more helpful and show increased affiliation towards imitating interaction partners (Duffy and Chartrand 2015). Likewise, rapid facial mimicry increases the duration of playful interactions in gelada baboons (Mancini et al. 2013a) and chimpanzees (Davila-Ross et al. 2011). Interestingly, in toddlers and monkeys, similar prosocial effects arise also when they are exposed to overt and systematic imitation. Indeed, toddlers are more helpful after being overtly imitated (Carpenter et al. 2013), while macaque (*Macaca nemestrina,* Paukner et al. 2005) and capuchin monkeys (*Sapajus apella*, Paukner et al. 2009) show increased levels of attention, interactivity and affiliation.

While the responses produced by the monkeys suggest that they implicitly discriminate being imitated from other types of interactions, toddlers and great apes show explicit imitation recognition, as they respond with so-called testing behaviours (e.g. Nielsen et al. 2005; Haun and Call 2008; Pope et al. 2015). Often these take the form of ‘behavioural repetitions’, i.e. the subject repeats a behaviour previously imitated by the experimenter. Such responses are regarded as active tests of the contingent correspondence between subjects’ own actions and those of the experimenter, and thus as indicating that apes and toddlers are aware of both action correspondence and the social causality involved in such imitative interactions (e.g. Nielsen et al. 2005; Bates and Byrne 2010). In other words, they are seemingly aware of the impact of their own actions on others’ behaviour (Bates and Byrne 2010) and also grasp the experimenter’s intention to imitate (Whiten and Suddendorf 2001) or, at least, to engage socially. In line with this interpretation, imitation recognition appears to be related to general social communicative skills. For example, chimpanzees that are more responsive to being imitated, also score higher on gesture production and comprehension, joint attention and gaze following (Pope et al. 2015).

Finally, the social conformism found for chimpanzees in some imitation learning tasks can arguably constitute further evidence of the proactive social function of imitation. More specifically, the chimpanzees have been found to copy the solution employed by other group members, even when this is less efficient or it goes against individual preferences. It has been proposed that such tendencies are an expression of conformity and benefit group cohesion (e.g. Hopper et al. 2011 and references therein).

In the present study, we focus on a kind of communicative imitation that has been described as the main mode of peer communication among pre-verbal toddlers (e.g. Eckerman et al. 1989; Eckerman and Stein 1990; Cordonier and Deschenaux 2014). This is characterised by the intentional, overt and immediate copying of predominantly familiar play actions and is accompanied by communicative behaviours, such as gaze contact and smiling (e.g. Cordonier and Deschenaux 2014). By means of such overt imitation, young children initiate and maintain interaction for significantly longer than in the absence of imitation (e.g. Eckerman and Stein 1990). In human ontogeny, this intentional form of communicative imitation peaks at the age of 18–24 months, thus preceding the emergence of nonconscious mimicry.

To our knowledge, this form of communicative imitation has not been previously studied in great apes, presumably because spontaneous imitation is suggested to be rare among apes (e.g. Call 2001), and evidence of familiar-action imitation is currently scarce (e.g. Tennie et al. 2012; Amici et al. 2014). Nevertheless, spontaneous imitation has been attested in great apes, both in captivity and in the wild, in inter- (e.g. Russon and Galdikas 1995; Byrne and Tanner 2006) and intraspecific contexts (e.g. Russon and Galdikas 1995; Hobaiter and Byrne 2010). It is often pointed out that apes’ spontaneous imitation can vary greatly with respect to fidelity to the modelled actions (e.g. Russon and Galdikas 1995; Byrne and Tanner 2006). This is also highlighted in studies in which apes, after extensive training with food reinforcement, learn to imitate on command (e.g. Custance et al. 1995; Call 2001; Carrasco et al. 2009). Nevertheless, such variation in imitation precision is present in toddlers as well, and, in the context of communicative imitation, ‘producing an exact match of an observed action is secondary to the goal of maintaining the interaction’ (Užgiris et al. 1989).

With respect to familiar-action imitation, recent experimental attempts at documenting it have yielded mainly negative results. In an imitation learning study by Tennie et al. (2012), only one non-enculturated chimpanzee showed evidence of familiar-action imitation. In another study involving zoo housed apes (Amici et al. 2014), none of the subjects imitated voluntary gestures (hand closing, wrist shaking), or other involuntary gestures than yawning (scratching, nose wiping). However, the imitation of familiar actions remains an understudied topic in nonhuman primates, thus it is possible that its frequency is underestimated^2^. Thus far, great apes have not been reported to imitate for seemingly social–communicative purposes. When imitated by humans, however, they often repeat the imitated action. It is unclear if this response is merely aimed at testing the ‘social mirror’ or if it qualifies as returned imitation aimed at maintaining the interaction, as argued for toddlers’ communicative imitation (e.g. Eckerman and Stein 1990). Arguing for the latter interpretation is the fact that imitation recognition appears to be related to social–communicative skills in chimpanzees (Pope et al. 2015).

Since imitation outside of learning tasks, i.e. the spontaneous imitation of familiar actions in social interaction, is underrepresented in the animal literature, the present study is exploratory and asks several questions:

1. Is spontaneous cross-species imitation present in both species and, if so, is its extent comparable across species?
2. Does spontaneous imitation appear to accomplish a social–communicative function in cross-species interaction?
3. Is the quality of imitation, in terms of imitative precision, comparable across species?
4. Do imitation rates, quality and repertoires vary across individual subjects (chimpanzees) or subject categories (visitors)?
5. Does proximity to the interaction partner influence imitation, considering that the topography of the site implies data collection in both distal and proximal interaction settings?
6. Do chimpanzees show evidence of imitation recognition in a context in which instances of being imitated are fleeting and immersed in a constant flow of diverse and distracting activities, as opposed to experimental settings where they are exposed to systematic, insistent and exaggerated imitation, usually by a familiar experimenter?

Finally, it is important to stress that obtaining data on the visitors’ imitative behaviours was equally important as collecting chimpanzee data. Indeed, studies on human spontaneous imitation in the context of cross-species interaction are lacking, yet such research is highly relevant for comparative purposes.

## Methods

### Subjects and site

The subjects were the five chimpanzees housed at Furuvik Zoo (Sweden)/Lund University Primate Research Station Furuvik in 2013, and the zoo visitors that attempted to interact with the chimpanzees or were the apparent targets of chimpanzee actions. At the time of data collection, the five chimpanzees formed a single non-reproductive group composed of one adult male (AM; 35 years old) and four females: one adult (AF; 29 years old), two subadult (SF1; 13 years old; SF2, 9 years old) and one juvenile (JF; 5 years old). With the exception of SF1, raised by its mother in captivity, the chimpanzees had mixed rearing (see Online Resource 1 for more details). The chimpanzee exhibit comprises indoor quarters and an outdoor area. Two of the indoor rooms are display areas. Here, visitors and chimpanzees can interact closely through glass. The outdoor area comprises an island with features such as trees, logs, platforms and caves, surrounded by a moat. The moat together with a fenced-off land strip on the visitor side of the moat places a distance of at least 6 m between visitors and chimpanzees (See Online Resource 1 for an aerial photograph of the outdoor enclosure).

### Data collection

Data collection—in total 52 observation hours—was conducted at Furuvik Zoo during a 21-day period, between June and August 2013. Data collection was generally conducted between 10 a.m. and 3 p.m., when the chimpanzee exhibit was most attended by visitors, and was always paused during public feeding, i.e. typically between 11:30 and 12:15. Data were recorded using pen and paper, since video recording of human participants in a public setting is only possible with prior consent. A video procedure would have thus entailed making visitors aware of the aims of the study, which in turn conflicted with the purposes of collecting data on spontaneous cross-species interactions. Filming only the chimpanzees while simultaneously collecting data on visitors’ behaviour by means of dictation was also too conspicuous.

Given good visibility conditions at the site, the small chimpanzee sample, and the salience of recorded behaviours, an all-occurrence sampling approach based on a predefined ethogram (see Online Resource 1) was used (Altmann 1974). To be able to establish both the presence and the extent of imitation, data collection focused on the behaviours that occurred during visitor–chimpanzee interaction. In addition, we recorded the interacting individuals. Visitors were categorized by gross age category (toddler, juvenile, subadult, adult) and sex (female, male, undetermined). Additionally, the category ‘crowd’ was used to capture those situations in which several visitors simultaneously showed an action.

Two observers, with extensive experience with the chimpanzees, collected data on separate days (T. P., 15 days; G. S., 6 days). The observers met before the start of the study to establish the ethogram and protocols for data collection (see Online Resource 1 for additional details). With respect to the latter, it was established that the observer’s priority was to follow the apes with the highest level of activity and thus highest potential to invoke the interactive interest of visitors. This was most relevant when the apes split into subgroups, which was rare. Data collection began as soon as a visitor or chimpanzee attempted to interact with the other species and continued for as long as the species responded to each other. When either species stopped directing behaviours towards the other, data collection stopped. The length of an interaction episode ranged from one action (when attempts to engage the other species failed) to as many actions as the two species directed at one another.

### Criteria for imitative actions

Each episode was screened separately for the presence of imitation, i.e. for situations in which the perception of a behaviour in another individual (the ‘model’) had arguably caused (i.e. was followed by) a similar behaviour in the observer (or ‘imitator’). This is a minimal requirement, and a feature shared by the various types of imitation proposed in the literature (e.g. Nielsen 2009), regardless of their purported functions or underlying mechanisms. For identifying instances of imitation, match (degree of similarity), contingency and directionality criteria were established.

#### Degree of similarity

Following a customary approach in imitation research, we distinguished between exact and partial imitation (e.g. Call 2001 and references therein), and both types of imitation were included in the analysis. All instances in which the imitator produced an action that was similar to a model’s action in terms of structural and dynamic features, and the involved body part, were coded as ‘exact imitation’. If the imitator responded with a similar action, that was performed with the same body part, but differed in some dynamic or structural feature, we coded the action as ‘partial imitation’. Consider, for instance, pressing the window with the open palm in response to a hit on the window with the open palm. In this example, the two behaviours are structurally similar and involve the same body part and object, but vary in terms of their dynamic features. Likewise, when a hit on the window with open palm was responded to with a fisted hit (‘knock’), the structural features of the two actions are different while everything else remains equal.

#### Contingency

To qualify as imitative, an action had to directly follow a modelled one and occur within the same episode. Although this entailed no further action being interposed between the modelled and copied actions, we allowed that: (1) the imitator completed ongoing actions; (2) the model, or another individual, interjected other actions, but usually in distinct modalities (e.g. a visitor claps hands, waves and calls the chimpanzee and the latter responds by clapping hands). In addition, it was important to preclude that subjects (visitors, chimpanzees) were not already producing that behaviour independently of the model. Consequently, to be coded as imitation, a behaviour should not have been produced by the imitator in the course of the previous episode or the previous 3 min (see also Amici et al. 2014 for a similar criterion).

#### Directionality

To qualify as imitation and as intentional, a matched behaviour had to be directed back at the model—i.e. not performed privately, or directed at another individual. Directedness toward a specific receiver is the main accepted behavioural criterion for intentional communication (e.g. Hobaiter and Byrne 2011). This criterion thus entails a responsive and engaged subject who is actively involved in and adjusts his/her own behaviour to a communicative interaction, as opposed to a subject that is a passive behavioural ‘reflector’ of perceived actions.

The chimpanzees’ responses to being imitated by visitors were also classified into three broad response categories: returned imitation (exact or partial), novel actions, or did not respond. Finally, the data was screened for potential indications of so-called imitative games. Characterized by a symmetric turn-taking usually focused on one action, such games are an important feature of toddlers’ communicative imitation and are regarded as an expression of mutual engagement, whereby each returned imitation also communicates a request to continue the exchange (Eckerman and Stein 1990; Eckerman et al. 1989). The minimal criterion for an imitative game is set at two turns per interacting individual (Eckerman et al. 1989). The current view is that, although apes recognize when they are being imitated, their responses never acquire such game-like features (Nielsen 2009).

## Results

### Question 1: the extent of imitation in the two species

In total, 3794 observations were made, of which 58% (*n* = 2211) were visitor actions and 42% (*n* = 1579) were chimpanzee actions (for additional results on interaction rates see Online Resource 2). In four additional cases it was unclear whether the agent was a human or a chimpanzee. Inter-observer agreement based on 521 additional observations conducted simultaneously by the two observers was very high: *k* = 0.843, SE = 0.013 (see Online Resource 2 for details). The 3794 actions constituted 974 episodes, of which 36% (*n* = 354) were bidirectional, involving actions performed by both species, in a turn-taking manner. Fifty-six episodes could not be classified with certainty as either uni- or bidirectional. Imitative actions were attested in both species in 37% (*n* = 132) of all bidirectional episodes, and in 11% (*n* = 6) of the episodes that could not be classified with respect to bi-directionality, for a total of 356 occurrences (repetitions excluded). To compare cross-species differences with respect to imitation rate and imitative precision, *z*-tests of proportions were employed. There was no difference between the species in terms of the extent of cross-species imitation (*Z* = 0.04, *P* = 0.97), as 9.37% (*n* = 148) of all actions performed by the chimpanzees and 9.41% (*n* = 208) of all actions performed by the visitors were classified as imitation.

### Question 2: the facilitating effect of imitation on social interaction

To determine if imitation had a facilitating effect on cross-species interaction by contributing to maintaining longer and more engaged interactions, episode length (i.e. number of recorded actions) was compared between episodes with and without imitation using the Mann–Whitney *U*-test. All unidirectional and unclassifiable episodes (*n* = 620) were excluded from this analysis. Significantly more actions (*U* = 7877.50, *Z* = −7.33, *P* < 0.001; median 1 = 7, median 2 = 4) were found in bidirectional episodes that included imitation (*n* = 132), as opposed to those that did not include imitation (*n* = 222).

### Question 3: the precision of imitation in the two species

With respect to imitative precision, exact imitation (as opposed to partial imitation) was observed significantly more often (*Z* = 2.93, *P* = 0.003) in humans (73% of all imitative actions, *n* = 151) than in chimpanzees (58% of all imitative actions, *n* = 86). There were primarily two types of actions that gave rise to partial imitations: manual actions on windows (hit, knock, press, stroke), and kiss-like gestures (knocking or pressing window with lips).

### Question 4: variations in the extent, quality and repertoires of imitation across individual chimpanzees and visitor categories

Four of the five chimpanzees produced imitative actions, the exception being SF1, who hardly interacted with visitors. A 2 × 4 χ^2^-test was conducted to compare the imitation rates of the four imitating individuals. This revealed a significant relationship between the presence of imitation and imitator identity (χ^2^ = 17.25, *df* = 3, *P* < 0.001; see also Table 1 for more details). Separate comparisons based on the *z*-test for proportions showed significantly higher imitation rates (all *P*s < 0.001) for AM (10.6%, *n* = 114) and SF2 (15.2%, *n* = 16) compared to AF (2.8% *n* = 5). The latter also imitated less than JF (6.4%, *n* = 13), but this difference was non significant (*Z* = 1.66, *P* = 0.097). In turn, the imitation rate of JF was significantly lower than that of SF2 (*Z* = 2.52, *P* = 0.012), and marginally lower than that of AM (*Z* = 1.85, *P* = 0.06). Partial imitation occurred significantly more often (*Z* = 0, *P* < 0.001) in the younger (79%, *n* = 23) than in the adult chimpanzees (33%, *n* = 44).

**Table 1:** Number of actions, imitation rates, and imitative precision in the outdoor and indoor environments for the individual chimpanzees and visitor categories

Subject/subject category	Outdoors					Indoors				
	No. actions	No. imitations	Imitation rate %	No. exact imitations	No. partial imitations	No. actions	No. imitations	Imitation rate %	No. exact imitations	No. partial imitations
Chimpanzees										
AF	171	5	3	5	0	8	0	0	0	0
SF1	13	0	0	0	0	4	0	0	0	0
SF2	25	0	0	0	0	80	16	20	3	13
JF	52	0	0	0	0	151	13	9	3	10
AM	527	32	6	32	0	546	82	15	43	39
Total chimpanzees	789^a^	37	5	37	0	790^a^	111	14	49	62
Visitor*s*										
AF	241	22	9	22	0	246	27	11	18	9
AM	243	22	9	20	2	179	13	7	7	6
SF	50	2	4	2	0	40	4	10	1	3
SM	33	7	21	6	1	36	4	11	3	1
JF	165	6	4	6	0	203	19	9	14	5
JM	157	10	6	10	0	170	20	12	14	6
TF	24	1	4	1	0	63	9	14	3	6
TM	32	0	0	0	0	72	12	17	3	9
Visitor crowd	124	17	14	17	0	81	13	16	4	9
Total visitors	1108^b^	87	8	84	3	1103^c^	121	11	67	54

*A* Adult, *F* female, *S* subadult, *J* juvenile, *M* male, *T* toddler,* Visitor crowd* several visitors simultaneously showed an action

^a^Including an additional case in which it was not possible to correctly identify the acting chimpanzee

^b^Including 39 additional cases in which it was not possible to correctly identify the acting visitor (28 cases) or visitor gender (11 cases)

^c^Including 13 additional cases in which it was not possible to correctly identify the acting visitor (two cases) or visitor gender (11 cases)

To determine if imitation rates differed among visitor categories (see Table 1), a 2 × 8 χ^2^-test was conducted on a data subset that included all data collected for the eight sex-age categories, excluding the collective category Crowd. The result of this test was non-significant (χ^*2*^ = 9.14, *df* = 7, *P* = 0.243). Imitative precision, on the other hand, was significantly related to age (χ^2^ = 22.41, *df* = 3, *P* < 0.001), with partial imitation occurring more often among toddlers (68% of all imitative actions, *n* = 15) than among juveniles (20%, *n* = 11), subadults (29%, *n* = 6) or adults (20%, *n* = 17).

Overall, the imitative repertoire of the chimpanzees included 14 distinct actions (Table 2). AM exhibited most flexibility, and his imitation repertoire (13 distinct actions) almost equated the cumulative repertoire of the group. The females imitated two to five action types each. The cumulated imitative repertoire of the visitors (Table 2) was larger, including—besides the 14 actions imitated by the chimpanzees—nine additional behaviours. Almost all of these additional behaviours were postures (e.g. body hugging, thumb sucking), or acts with a physiological function (e.g. yawning, scratching). Among visitors, the imitative repertoire of toddlers (five distinct actions of which three were shown by males and all five by females) was lower than that of adults (15 actions; 12 of which were shown by males and 11 by females), subadults (11 actions; seven of which were shown by males and six by females) and juveniles (14 actions; all 14 of which were shown by males and seven by females).

**Table 2:** Imitative repertoires for visitors and chimpanzees

Imitated action	Actions imitated by the visitors			Actions imitated by the chimpanzees		
	Exact imitation	Partial imitation	Total	Exact imitation	Partial imitation	Total
Pressing lips to window	25	1	26	25	16	41
Pressing window with hand	1	5	6	3	26	29
Knocking window with hand	21	22	43	15	5	20
Clapping hands	47	0	47	20	0	20
Stroking window with hand	1	1	2	0	11	11
Knocking head with hand	14	0	14	8	0	8
Hitting window with hand	2	12	14	2	4	6
Body swaying	3	0	3	5	0	5
Object throwing	6	0	6	3	0	3
Leaning forward	0	0	0	1	0	1
Begging	2	0	2	1	0	1
Extending arm	2	0	2	1	0	1
Pouting lips	3	0	3	1	0	1
Head bobbing	4	0	4	1	0	1
Body picking	1	0	1	0	0	0
Body shaking	1	0	1	0	0	0
Waving	1	0	1	0	0	0
Quick approach	1	0	1	0	0	0
Yawning	0	3	3	0	0	0
Self-hugging	3	0	3	0	0	0
Body scratching	4	0	4	0	0	0
Resting head on hand	4	0	4	0	0	0
Thumb sucking	5	0	5	0	0	0
Knocking window with lips	0	13	13	0	0	0
Total imitated actions	151	57	208	86	62	148

Imitated actions are listed in order of frequency with which they were imitated by the chimpanzees, and then by the visitors

Only a few actions were frequently imitated by either species. These coincided across species and included hand clapping and actions on the windows (see Table 2). Both species imitated primarily actions that were already in their behavioural repertoire.

### Question 5: the influence of interactional proximity on imitation

To assess if presence and precision of imitation was influenced by proximity to the interaction partner, and whether these potential influences were differentially expressed across species, two log linear analyses were conducted. Interactional proximity was defined by two values: distal interaction, i.e. when the species interacted across the moat or high wall (outdoors), and proximal interaction when the interaction took place through glass (indoors).

The first analysis focused on determining if interactional proximity affected the extent of imitation in the two species, and included the following factors: proximity (outdoors, indoors); presence of imitation (present, absent); and species (*Homo sapiens*, *Pan troglodytes*). The analysis produced a final model that retained the highest level effect, indicating a three-way significant interaction between presence of imitation × species × proximity (χ^2^ *=* 11.840, *P* = 0.001). The likelihood ratio for this model was χ^2^(0) = 0, *P* = 1, suggesting it to be a perfect fit for the observed data. To break down this interaction effect, χ^2^ analyses were conducted separately for each species which revealed a significant relationship between proximity and presence of imitation. Both species produced more imitative actions indoors than outdoors (chimpanzees, χ^2^ = 40.72, *df* = 1, *P* < 0.001; visitors, χ^2^ *=* 6.31, *df* = 1, *P* = 0.012; see also Table 1).

The second log linear analysis aimed at establishing interactional proximity proximity affected imitative precision, and included the following factors: imitative precision (exact imitation, partial imitation); species (*H. sapiens*,* P. troglodytes*), and proximity (outdoors, indoors). This analysis produced a model that retained, at the highest level, two two-way interaction effects: proximity × species (χ^2^ = 4.99, *P* = 0.025), and proximity × imitative precision (χ^2^ = 97.76, *P* < 0.001). A likelihood ratio test showed that this model was an acceptably good fit for the data (χ^2^ = 5.08, *df* = 2, *P* = 0.08). The first interaction has been discussed in the previous paragraph, whereby imitation by both species was found to be more frequent in indoor than outdoor locations. χ^2^-tests conducted to follow up on the second interaction revealed that partial imitation by either species was significantly more frequent indoors (chimpanzees, χ^2^ = 35.57, *df* = 1, *P* < 0.001; visitors, χ^2^ = 43.14, *df* = 1, *P* < 0.001). Indoors, the distribution of partial and exact imitation was not significantly different between chimpanzees and humans (χ^2^ = 2.92, *df* = 1, *P* = 0.08).

Among the chimpanzees, only AM engaged in imitative interactions both indoors and outdoors. The females imitated (and predominantly acted) either exclusively outdoors (AF) or indoors (SF2, JF). Likewise, the youngest visitor category (toddlers) imitated (and predominantly acted) indoors (Table 1).

### Question 6: chimpanzees’ responses to being imitated

Of the 1579 actions produced by the chimpanzees, 196 were imitated by humans. Chimpanzees’ responses to being imitated included 71 instances of returned imitations, and 37 instances of non-imitative actions. In four additional cases it was not possible to establish if a chimpanzee responded with returned imitation or another action. Most responses to being imitated were single actions, as opposed to action sequences (of typically two, but up to seven actions), which were found in 10.95% of instances. Whether the chimpanzees responded or not, was not related to the type of imitation (exact or partial) to which they were exposed (χ^2^ = 0.51, *df* = 1, *P* = 0.52).

In 42 imitative exchanges the chimpanzees performed the minimal two turns required by the imitation game criterion, and all four imitating chimpanzees engaged at least once in such imitative games. Of these occasions, 11 were extended imitative exchanges, involving at least three—but up to ten—turns by the chimpanzee. Besides these 42 exchanges, we recorded an additional episode, which was difficult to fit to our restrictive contingency criterion, but which unfolded as a prolonged imitation game lasting as long as 10 min. This interaction consisted primarily of a sequence of mutual imitations of hand clapping and body rocking with arms slightly lifted in front of the body (the chimpanzee) or at shoulder height (the visitor). For the chimpanzee, play signals (play face, ground slaps) were observed to co-occur with imitation in the course of this interaction. Since the visitor appeared to be a tourist from abroad, it is unlikely that an interactional history existed between her and the chimpanzee.

No response was recorded in 84 cases (see Online Resource 2 for likely causes of a lack of response). Interestingly, chimpanzees appeared unlikely to respond when visitors imitated bodily postures or actions with physiological functions, such as yawning, scratching, etc. (two potential responses in 19 cases; *P* < 0.001, binomial test).

## Discussion

To our knowledge, the present study is the first to systematically investigate spontaneous imitation in free interactions between zoo visitors and zoo-housed chimpanzees. Previous visitor studies have found, in general, little interaction between *H. sapiens* and* P. troglodytes*. Moreover, visitor-directed chimpanzee actions appeared to have been generally unrelated to visitors’ behaviours unless food was involved, which elicited begging behaviours (e.g. Cook and Hosey 1995; Wood 1998). As suggested by reviewers, factors that might account for such different results are exhibit design and animal husbandry practices. The presence of large glass walls might facilitate close proximity between the species and thus higher levels of interaction. With respect to husbandry practices, zoo animals that are engaged by keepers in social interactions subsequently respond more positively to unfamiliar visitors (see Hosey and Melfi 2015).

In this study, during 52 h of observation, we recorded 354 episodes of cross-species reciprocal interactions. Food-related gestures were exclusively performed by AF. Imitative actions by one species or by both occurred in 37% of the 354 reciprocal interactions, and had an effect of prolonging and increasing engagement in the interaction. Indeed, episodes that included imitation contained twice as many actions as those without imitation. For both species, all imitated actions were familiar ones. This supports the view that, for a scientific understanding of the roles of behaviour copying, studies of imitation should not only be limited to the area of social learning.

The imitative responses documented here did not exhibit the characteristics of reflexive responding. On the contrary, several of their features suggest intentional communication as both species produced ‘targeted’ imitations, i.e. while visually and bodily oriented towards the model and while monitoring the interaction. Directedness toward a specific receiver and sensitivity to attentional stances are the most commonly accepted behavioural criteria for intentional communication in apes (e.g. Hobaiter and Byrne 2011). It has been argued, however, that apes’ intentionality in communicative situations lacks a crucial ‘shared’ aspect, which in humans supports the motivation to share experiences with others by means of communication (e.g. Carpenter and Call 2009; Nielsen 2009). As a consequence, these authors have proposed that, in apes, imitative exchanges might lack the communicative component. For example, in imitation learning tasks, apes are found to engage in shorter bouts of communicative behaviours (joint attention, gazing at the model’s face) than children (e.g. Carpenter and Tomasello 1995). However, it has also been found that communicative imitation in children is favoured by non-learning contexts, and that communicative behaviours decrease or even vanish in instrumental imitation learning tasks (Cordonier and Deschenaux 2014). With respect to imitation recognition, previously tested apes have not been reported to show signs of enjoyment and playfulness, e.g. to engage in imitative games (Nielsen 2009). The evidence in our study suggests, however, that apes can show playfulness in imitative contexts and that imitation might serve a communicative purpose. We base this conclusion on the fact that, on several occasions, the chimpanzees engaged in prolonged imitative exchanges that satisfy the criteria to qualify as imitative games. Additionally, we observed that, at least on some occasions, AM exhibited play face expressions in the context of imitative exchanges. We therefore propose that the imitative episodes described here are reminiscent of toddlers’ communicative imitation. In such contexts familiar actions are overtly imitated for initiating and maintaining interaction, resulting in mutually rewarding and sustained engagement in the interaction, occasionally leading to imitation games (Eckerman et al. 1989). This is in agreement with the suggestion that apes exhibit signs of shared intentionality in the context of social games, especially considering that in at least three documented cases—two with bonobos (Pika and Zuberbühler 2008) and one with gorillas (Tanner and Byrne 2010)—the social game involved symmetrical object exchanges, and thus could have been potentially imitative in nature.

Further argumenting for the intentional and communicative character of the imitation documented in this study is evidence of flexibility and selectivity. Interestingly, the most flexible chimpanzee exhibited an imitative repertoire of a similar size to that of the most imitative visitor categories (adults, subadults and juveniles). On the other hand, only few actions were preferentially selected for seemingly imitative–communicative purposes by both species. This selectivity is likely the result of a long interactional history between visitors and chimpanzees that—among other things—allowed the chimpanzees to learn the effect that copying specific actions has on visitors. Such an interpretation is plausible, considering that apes show selectivity for behaviours experienced as communicatively efficient (Hobaiter and Byrne 2011). Even if repeated interaction had a reinforcing role in shaping chimpanzees’ imitative responses, it is worth emphasizing that the only plausible reinforcement was the rewarding nature of (continued) social interaction itself. While zoo staff uses food incentives in many interactions with the chimpanzees, in our experience these interactions do not involve imitation, and are thus not likely to reinforce such responses. They more likely result in begging gestures, which we do see directed at zoo visitors as well. The actions we observed in imitative games, however, were typically not ritualized begging gestures.

Another finding is that, in both species, imitation rate increased in the indoor environment, suggesting that physical proximity plays a role in facilitating imitation. In both species, physical proximity further coincided with increased partial imitation; conversely, exact imitation dominated almost exclusively in distal imitative exchanges. It is unclear, however, whether such results are due to higher requirements for precision in distal communication, or by the different repertoires of imitated actions indoors and outdoors. For example, Call (2001) found an orangutan subject to be more successful at matching actions performed with gross body parts than those involving smaller parts, e.g. a raise-finger action was mimicked with a raise-arm action. Thus the almost exclusive predominance of exact imitation in the outdoor locations could be related to the fact that larger gestures and gross motor patterns were more likely to be performed and imitated in that environment. In the indoor locations, on the other hand, imitated actions involved more detailed matching, such as, for example, knocking on the window with knuckles or palm. In any case, it has to be stressed that this increase in partial imitation was similar in both species. Physical proximity, especially in cases when space can be shared, such as a windowpane, might be a crucial variable and requires further attention in imitation research. It is also possible, however, that glass surfaces (rather than proximity), afforded actions that facilitated imitation.

Besides cross-species commonalities there were also notable differences. For example, humans showed overall higher imitative precision. The data further indicate differences concerning the size of the cumulative repertoire of imitated behaviours, which was larger among visitors than chimpanzees. This can be due to the large difference in the number of individuals that represented each species, but also to the fact that—unlike humans—the chimpanzees did not imitate bodily postures or behaviours with a physiological function. In this respect, we found a potential parallelism with imitation recognition, i.e. how the chimpanzees responded when their behaviours were imitated by the visitors. The chimpanzees responded when visitors imitated their actions, but they were not observed to react when visitors imitated their postures or physiological behaviours (e.g. yawn, scratch). That chimpanzees failed to show a response when visitors copied their postures could be related to the generally non-conscious nature of this type of mirroring behaviour, which, as opposed to overt imitation, does not elicit behaviours indicative of explicit imitation recognition. Although recent studies highlight the similar positive social consequences of both overt imitation and nonconscious mimicry and regard them as related phenomena (e.g. Carpenter et al. 2013; Duffy and Chartrand 2015), further research is needed to specify the relationship between these imitative behaviours.

Further research should moreover be directed towards establishing how representative imitative communication is of chimpanzees’ intra- and cross-specific interactions, and if the present results can be replicated. Our data show that four of the five chimpanzees that were observed produced imitative actions in cross-species interactions. These results contrast with previous experimental studies that found familiar action imitation to be restricted to certain, ‘gifted’ individuals (Tennie et al. 2012), or to specific non-voluntary behaviours such as yawning (Amici et al. 2014). Since human-raised chimpanzees show a higher orientation towards human behaviour and better imitative skills (e.g. Byrne and Tanner 2006; Tennie et al. 2012 for discussions), rearing history is a potential candidate to explain these differences. In the case of this group, however, it is not possible to draw any firm conclusions. The only mother-reared chimpanzee (SF1) in the group interacted only minimally with visitors, while the four imitating chimpanzees, which had mixed rearing histories, exhibited variations in imitation rates. Interestingly, AF, the only chimpanzee in the group that was temporarily (i.e. for 18 months) raised in a human home without simultaneous contact with conspecifics, was also the chimpanzee that imitated the least. One of the chimpanzees (AM) was by far the most interactive individual in the group, as well as the most flexible imitator, which might support the view that certain ‘gifted’ individuals are more prone to imitation (Tennie et al. 2012). Additionally, imitation recognition in chimpanzees appears to be related to sex, as males are more responsive than females when being imitated by a human experimenter (Pope et al. 2015). Yet AM’s overall imitation rate was relatively similar to those of SF2 and JF. It thus remains to be established whether other factors can further account for a chimpanzee’s propensity for copying behaviour. Finally, the results reported could be attributed to the nature of the familiar actions performed in these imitative interactions, as well as to the context in which they were deployed. In our study, we capture the imitation of familiar communicative or play behaviours in the context of relaxed and positive social interactions. Moreover, food acquisition was not involved, unlike in previous studies.

With respect to visitors’ behaviour, subadult males were the most imitating category outdoors, while toddlers almost never imitated in this environment. Indoors, however, toddlers showed an increase in both interactivity and imitation rate, becoming the most imitating category in this proximal setting.

An advantage of using an observational approach is that we were able to simultaneously document a broader range of variables than is possible in an experimental setting, while capturing spontaneous occurrences in a naturalistic setting. Inherent limitations to this method (e.g. the inability to study potentially moderating variables in controlled conditions) limit, however, the conclusions which can be drawn from this study. Overall, the present study highlights the imitation of familiar behaviours in apes as a relevant research topic and provides testable hypotheses concerning its role in promoting and maintaining play and interaction, as well as concerning the potential role of proximity in promoting imitation. Our data add to previous observations of spontaneous imitation by apes and indicate that, unlike previously suggested, the chimpanzees’ imitative actions might serve a socio-communicative function by maintaining social interaction. Moreover, imitation seemed to take on the characteristics of play, giving rise to so-called imitation games. We hope that the results presented here will incite further inquiries into this topic.

## Electronic supplementary materials

Below is the link to the electronic supplementary material.

Supplementary material 1 (DOCX 438 kb)

Supplementary material 2 (DOCX 17 kb)

## Footnotes

1 For recent reviews on the learning function of imitation, see e.g. Carpenter and Call (2009); Bates and Byrne (2010); Hopper et al. (2011); Tennie et al. (2012).

2 It has been proposed that a more appropriate label for familiar-action imitation could be ‘response facilitation,’ as this form of imitation can be construed as a case of priming (Byrne and Tanner 2006; Bates and Byrne 2010). In other words, copying an observed action is facilitated by the activation of pre-existing (neural) records of that (or a similar) action. In this study, we do not employ this terminology, since the imitation of familiar actions is not a unitary phenomenon. Extant evidence suggests the need to distinguish at least between the nonconscious mimicry of primarily involuntary actions and the intentional (and communicative) imitation of actions that are more under voluntary control, since both shared and distinct underlying mechanisms subserve these forms of imitation (e.g. Amici et al. 2014).
