# Supplementary material for: Spontaneous cross-species imitation in interactions between chimpanzees and zoo visitors
Source: Primates. 2017 Aug 16;59(1):19–29. doi: 10.1007/s10329-017-0624-9 (PMC5740201; doi:10.1007/s10329-017-0624-9)
Supplement: Supplementary file 1 — Supplementary material 1 (DOCX 438 kb) [file 10329_2017_624_MOESM1_ESM.docx]

**Spontaneous cross-species imitation in interaction between chimpanzees and zoo visitors**

**Journal:** *Primates*

Authors: Persson T, Sauciuc GA, Madsen EA

Affiliation: Lund University, Department of Philosophy, Cognitive Science

E-mail address: [Gabriela-Alina.Sauciuc@lucs.lu.se](mailto:Gabriela-Alina.Sauciuc@lucs.lu.se)

**Online resource 1: Additional methodological details**

**Rearing history of the chimpanzee subjects**

The five chimpanzees that served as subjects in the study are genetically unrelated and, at the time of data collection, formed a single, non-reproductive group. The chimpanzees have transferred to Furuvik Zoo at various ages: the adult male (AM) and the older subadult female (SF1) were 5-years old, the adult female (AF) was 2 years, the younger subadult female (SF2) was 4-months and the juvenile female (JF) was only days old.

AF is wild-caught and was mother raised until the age of about 6-months. Thereafter, she has been raised in a human home before transferring to Furuvik Zoo, at 2 years of age. AF became foster mother to two of the females in the group: SF2 (orphaned at 4-months of age) and JF (rejected at birth). Before and during the adoption process, the two females were hand-raised by a keeper (IMP), while having daily contact with the chimpanzee group. Peculiar to these chimpanzees is their history of close interactions with IMP, who regularly used to spend time with them inside their enclosure. These interactions consisted primarily of mutual grooming and play (e.g. chasing, play-biting, etc.). It cannot be excluded, however, that imitative actions occurred in the history of their interaction. The chimpanzees’ interactions with other keepers included usual husbandry and social practices (feeding, cage shifting, etc.). Prior to the observation period, the chimpanzees had participated in several cognitive experiments, none of which involved imitation.

**Ethogram and behaviour recording procedures**

The ethogram has been jointly established by the first two authors of this study, who were also involved in the data collection as observers. To derive the inventory of behaviours to be observed, they relied on an existing ethogram at the research station used for general observation, on previous studies that documented interactions between zoo visitors and chimpanzees (e.g. Cook and Hosey 1995; Wood 1998) and on their previous experience with the chimpanzee subjects included in this study and visitor behaviour at this exhibit. Based on these sources, a list of potential behaviours of interest was obtained. Subsequently, the observers met prior to the start of data collection to agree on procedures and criteria. The onset of each new bout of interaction was marked by a timestamp (i.e. hour and minute of the day when the interaction started), and this was employed for delimiting interaction episodes. Data collection was paused when the individuals involved in the interaction ceased to direct actions at each other for at least 30 seconds. The start and end of an entire session of data collection were also marked by a timestamp.

The observer’s priority was to follow the animals with highest level of interaction. In practice, since the animals only seldom separated, most often this resulted in having the whole group of 5 chimpanzees under observation. In case a new bout of cross-species interaction, involving another chimpanzee, would begin at a different location, while the observer was engaged in recording an ongoing interaction, it was established that the observer should ignore the new interaction.

The observer positioned her/himself so that s/he could simultaneously observe both the animals and the visitors that faced them. In a number of cases, we weren’t able to establish the initiator of an interaction, and all these cases have been recorded as such, i.e. initiator ‘unknown’. Behaviours were recorded action by action, at two levels of specification. Each action was described in terms of a generic class (vocalization, gesture etc.) and a specifying complement (e.g. shout, wave). Whenever judged necessary, additional comments were added to each record, e.g. occasional facial expressions of emotions, which were not part of the ethogram. Such comments were not treated as systematic data in subsequent analysis. At the end of each observation day the observer transcribed and digitalised the notes.


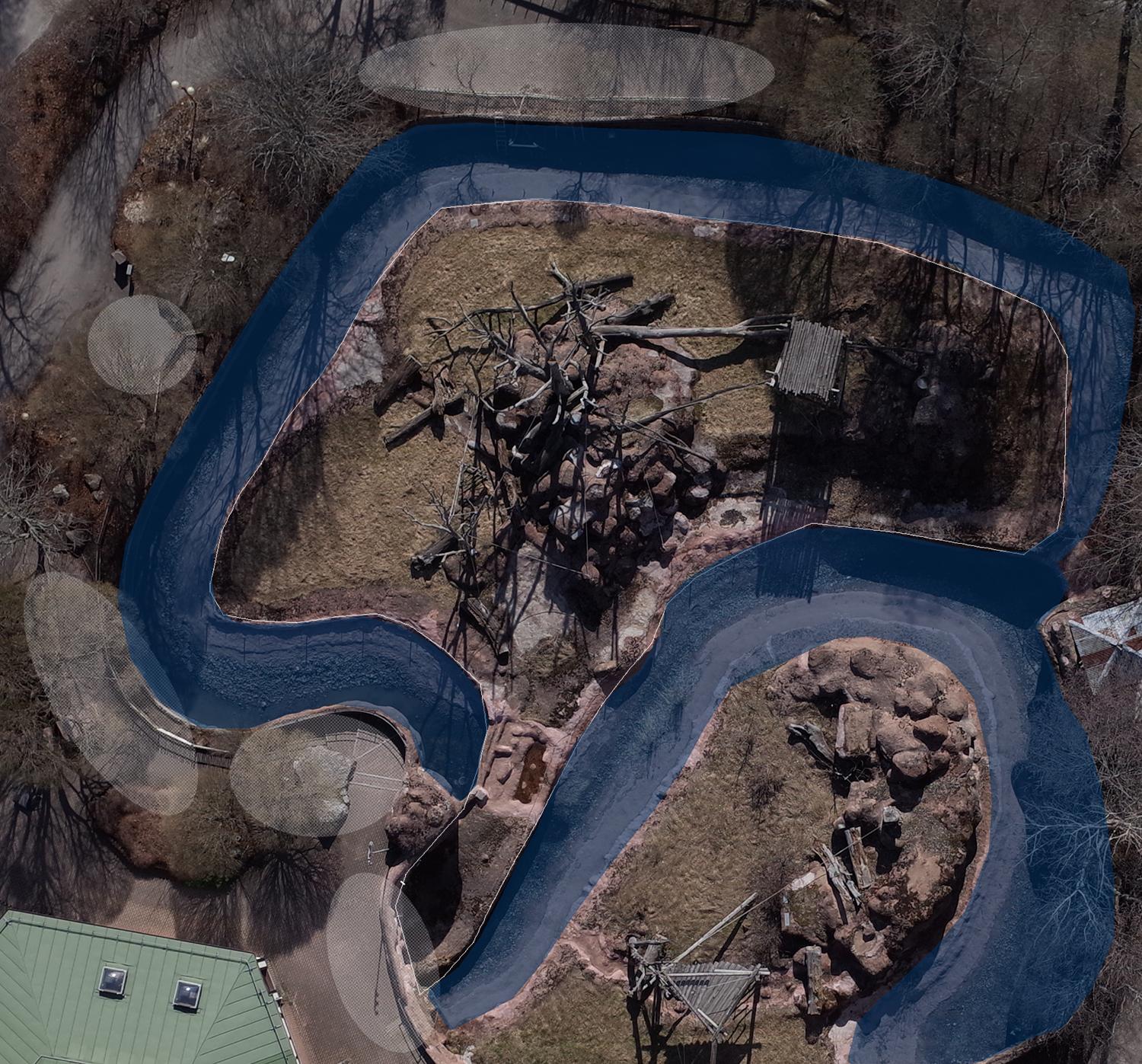


Aerial photograph of the outdoor enclosure (© Metria and University of Gävle). The image has been edited by the authors to highlight the five areas from which visitors could watch the chimpanzees and from where data were recorded. Blue colour to the water moat has been added.

| List of frequently occurring behaviors included in the ethogram. Generic action categories (first column of the table) can be flexibly combined with relevant specifications (last two columns of the table). | | |
| --- | --- | --- |
| GENERIC ACTIONS | ACTION SPECIFICATION | |
| ‘Atypical’ behaviour, often specific to one or two individuals  Body manipulation  Clapping  Facial expression, when particularly salient  Gesture performed with hand or arm  Hitting action  Knocking action  Object manipulation  Positioning  Pressing of hand or other body part against a surface  Stroking action (on object, surface, body part)  Style (primarily of locomotion)  Posture / trajectory  Vocalization  Not visible | ***Surfaces***  Window  Gras  Structure  Cement  Food item  Wood wool  Rock  Floor  ***Body parts***  Hand  Feet  Trunk  Head  Arm  Mouth  Lips  ***Posture / Trajectory***  Approach  Backside  Chase  Down  Head-in-hand  Hug body  Lean in  Leave  Presenting  Up  Viewing  ***Styles***  Aggressive  Swingingly | ***Gestures***  Beg  Extend arm  Head bobbing  Wave  ***Manipulations***  Scratch  Pick  Throw  ***Expressions***  Smile / laughter  Yawn  Gape  Raspberry  Pout  ***Vocalizations***  Whistling  Yelling  Ape sound  Addressing  ***‘Atypical’***  Knock head  Rubbing  Shaking  Thumb sucking  ***Other***  Miscellaneous gesturing, not captured by any of the above (behaviour is specified as comments)  ***Unclear*** |
