# Supplementary material for: Spontaneous cross-species imitation in interactions between chimpanzees and zoo visitors
Source: Primates. 2017 Aug 16;59(1):19–29. doi: 10.1007/s10329-017-0624-9 (PMC5740201; doi:10.1007/s10329-017-0624-9)
Supplement: Supplementary file 2 — Supplementary material 2 (DOCX 17 kb) [file 10329_2017_624_MOESM2_ESM.docx]

**Spontaneous cross-species imitation in interaction between chimpanzees and zoo visitors**

**Journal:** *Primates*

Authors: Persson T, Sauciuc GA, Madsen EA

Affiliation: Lund University, Department of Philosophy, Cognitive Science

E-mail address: [Gabriela-Alina.Sauciuc@lucs.lu.se](mailto:Gabriela-Alina.Sauciuc@lucs.lu.se)

**Online resource 2**

**Visitor statistics**

Although we could not record their exact number, the visitors that attempted to interact with the chimpanzees clearly constituted a minority of those that stopped by the chimpanzee exhibit. Since the chimpanzee exhibit is the first encountered by the visitors as they enter the park, visitor entry figures constitute a fairly good approximation of the number of visitors that attended the exhibit during the days of data collection. Across the period of data collection, a total number of 50 299 visitors entered the park (daily median = 1 953; range: 367 - 10 056 visitors / day).

**Overall number of observations in relation to visitor number, type of environment and part of the season**

In total, 3 794 observations were made, of which 58% (N=2 211) were visitor actions and 42% (N=1 579) were chimpanzee actions. In 4 additional cases it was unclear whether the agent was a human or a chimpanzee. The 3 794 actions constituted 974 episodes, of which 36% (N=354) were bidirectional, involving actions performed by both species, in a turn-taking manner. Fifty-six episodes could not be classified with certainty as either uni- or bidirectional. In the outdoors environment, where crowd size can vary considerably, chimpanzees’ level of cross-species interactivity (number of observed actions per/ minute) increased as visitor numbers increased (*r*=0.48, *P*=0.04, N=18). A similar variation was not found indoors, where space limitations constrain crowd sizes to remain stable (*r*=0.05, *P*=0.889, N=11). In the outdoors environment, cross-species activity levels declined significantly (*U*=13, *Z*=- 2.428, *P*=0.014) from the first part of the observation period (*Mdn=*1.4) towards the end of it (*Mdn*=0.6). A similar variation was not observed indoors (*U*=13.000, *Z*=-365, *P*=0.792).

**Inter-observer reliability**

Two approaches have been used for assessing interobserver reliability, by (1) comparing the observation rates of the two observers for the entire data set and (2) determining level of observation agreement based on an additional data set obtained by independent and simultaneous observation by the two observers.

*Comparison of observation rates.* Given the differences reported in the previous section, inter-observer reliability was assessed by comparing the observation rates (mean number of observations per minute) of the two observers separately for the two environments and observation periods. This was justified by the fact that one observer (GS) predominantly collected data during the first part of the observation period and that visitor number was also significantly higher when GS collected data compared to when TP collected data (*U*=18, *Z*=-2.102, *P*=0.036, *Mdn1*=3132, *Mdn2*=1660). No difference was found between the two observers in the indoor (*U*=7, *Z*=-471, *P*=0.8, *Mdn1*=2.2, *Mdn2*=1.8) or outdoor environment (*U*=3; *Z*=-1.715; *P*=0.11, *Mdn1*=1.6; *Mdn2*=1) for the first part of the observation period. For the second part of the observation period, only observation rate ranges were compared, as one observer collected data for one day only. These were similar: GS=0.7 observations per min. vs. TP, *M*=0.67 observations per minute (range 0.2 - 0.9 observations per minute).

*Comparison of level of agreement*. For determining the level of interobserver agreement with respect of recorded actions, an additional data set was collected by means of simultaneous and independent observation by the two observers. Data collection took place on four separate days, for a total of 9 hours. A total of 521 observations were recorded, capturing at least one instance for 43 action categories, based on the predefined ethogram. For the purpose of data analysis, the category ‘miss’ was added in order to code omissions by each of the observers. Thus the matrix for calculating interobserver agreement included 43 +1 columns and 43 + 1 rows. Interobserver agreement as calculated using Cohen’s kappa was found to be very high: k=0.843, SE=.013.

**Potential explanations for cases of ‘no response’ to being imitated**

For 76 of the 84 cases in which the chimpanzees did not respond to being imitated, we have observations of likely causes. For example, we recorded 18 cases of visitor ‘over-action’, i.e. one or more visitors responded by rapidly producing at least four different actions, besides the imitative response. In at least five cases, at the end of such over-action episodes, we could observe a returned imitation by the chimpanzee. Such late responses were, however, not validated, since they did not conform to our contingency criteria. In 10 other cases, various interferences hindered the continuation of interaction, such as loss of an involved object (2 cases), feeding start (1 case), visitors obstructing interactive access (1 case), and visitor’s attention redirected to another chimpanzee (6 cases). In 6 additional cases, the visitor imitated food-begging behaviours, while in 25 cases visitors’ imitation coincided with the end of the interaction*.* Finally, chimpanzees appeared unlikely to respond when visitors imitated bodily postures, or actions with physiological functions, such as yawning, scratching, etc. (2 potential responses in 19 cases, *P*<0.001, binomial test).
